# Proof of concept for aqueous two-phase system-based extraction of cell-free DNA from plasma for liquid biopsy applications

**DOI:** 10.1038/s41598-026-45585-z

**Published:** 2026-04-02

**Authors:** Rafaela Meutelet, Benedikt C. Buerfent, Timo Hess, Julia Teply-Szymanski, Paul Jank, Johannes Oldenburg, Heiko Rühl, Jürgen Hubbuch

**Affiliations:** 1 Institute of Process Engineering in Life Sciences, Section IV: Biomolecular Separation Engineering, Karlsruhe Institute of Technology, Karlsruhe, 76131 Germany; 2 BioEcho Life Sciences GmbH, Cologne, 50829 Germany; 3 Institute of Pathology, Philipps-University Marburg and University Hospital Marburg (UKGM), Marburg, Germany; 4 Department of Gynecology with Breast Center, Charité – Universitätsmedizin Berlin, corporate member of Freie Universität Berlin and Humboldt Universität zu Berlin, Berlin, 10117 Germany; 5 Institute for Experimental Hematology and Transfusion Medicine, University Hospital Bonn, Bonn, 53127 Germany; 6 Institute of Human Genetics, University of Bonn, Bonn, Germany

**Keywords:** Aqueous two-phase systems (ATPS), cfDNA extraction, DNA purification, Liquid biopsy, Next-generation sequencing, Biological techniques, Biotechnology, Molecular biology

## Abstract

Efficient extraction of circulating cell-free DNA (cfDNA) from plasma is critical for liquid biopsy applications, but remains challenging due to cfDNA’s short fragment size, low concentration, and the presence of interfering proteins and genomic DNA. This study presents a simple extraction process based on aqueous two-phase system (ATPS) capture and reverse elution purification as a potentially lysis-free alternative to adsorption-based methods. Short DNA fragments selectively partitioned into the salt-rich bottom phase, achieving up to 65% recovery after purification while removing 99.7% of plasma proteins. Reverse elution enabled efficient desalting and up to fourfold DNA concentration under mild, aqueous conditions. The extracts showed no qPCR inhibition and displayed a cfDNA-like size profile with a cutoff around 750 bp, indicating selective enrichment of short fragments. Although the total yield was slightly lower than that of the QIAamp Circulating Nucleic Acid Kit (QIAGEN), the ATPS workflow substantially reduced processing time, equipment needs, and cost. Next-generation sequencing of cfDNA reference material extracted from DNA-free plasma confirmed compatibility with library preparation and sequencing workflows, providing proof of concept for a simplified cfDNA extraction strategy for amplification-based liquid biopsy applications.

## Introduction

Molecular diagnostics based on nucleic acids have become central to precision medicine, enabling individualized disease characterization and treatment^1,2^. Cell-free DNA (cfDNA), referring to extracellular DNA fragments released into the bloodstream primarily through apoptosis and necrosis of cells, circulates in blood and other body fluids and has emerged as a key biomarker for liquid biopsy, offering a minimally invasive alternative to tissue biopsy^3,4^. Its analysis serves applications ranging from cancer management to prenatal testing and transplant monitoring^5–8^. In oncology, a small fraction of cfDNA originates from tumor cells and is referred to as circulating tumor DNA (ctDNA), which carries tumor-specific genetic alterations^9^.

However, the analysis of cfDNA remains challenging, mainly due to its low abundance in plasma^10^. Typically present at concentrations below 40 ng/mL, cfDNA consists of short, nucleosome-associated fragments around 167 bp that are rapidly degraded and easily lost during processing^11,12^. Because ctDNA represents only a minute fraction of total cfDNA, contamination with high–molecular–weight genomic DNA (gDNA) released from lysed blood cells during sample handling can compromise mutation detection and quantification^13^. Consequently, cfDNA extraction remains a critical bottleneck for sensitive and reproducible downstream molecular analyses, such as next-generation sequencing (NGS), as extraction efficiency and preservation of fragment size can significantly influence analytical outcomes^14–16^. These challenges are further amplified in clinical diagnostics, where rapid turnaround times and high throughput are required with limited sample volumes.

Commercial cfDNA extraction kits predominantly rely on solid-phase adsorption, most commonly using silica membranes or silica-coated magnetic beads under high-salt conditions^17^. In these systems, nucleic acids bind to the silica surface in the presence of chaotropic salts and are subsequently recovered through sequential washing and elution steps^18^. While these approaches provide high purity and are widely used as reference methods, such as the QIAamp Circulating Nucleic Acid Kit (QIAGEN), they present several practical and analytical limitations. The multi-step bind-wash-elute workflow is time-consuming, requires repeated manual handling and vacuum or centrifugation steps, and may increase the risk of cross-contamination^19^. Automated magnetic-bead-based systems can mitigate this risk and improve workflow consistency, but other limitations remain. For instance, incomplete removal of chaotropic salts may inhibit downstream polymerase chain reaction (PCR). In addition, because cfDNA is typically present at very low concentrations, incomplete adsorption or inefficient elution can directly reduce analytical sensitivity^20^. From an analytical perspective, the recovery of short DNA fragments may be less efficient, potentially introducing fragment-size bias and affecting the representation of cfDNA populations^14,21^. Furthermore, contamination with high–molecular–weight gDNA released during sample handling can compromise the detection of rare tumor-derived variants. Together with high reagent costs, limited throughput, and the need for specialized equipment, these factors highlight the need for simpler, scalable, and cost-effective extraction strategies. Consistent with this, comparative studies have reported substantial variability in cfDNA yield, purity, and fragment size distribution across manual and automated extraction workflows, underscoring that cfDNA isolation remains a critical and not yet fully standardized pre-analytical step in liquid biopsy analysis^17,22–24^.

Given these challenges, liquid–liquid extraction using aqueous two-phase systems (ATPS) offers a promising alternative to conventional solid-phase approaches. ATPS enable biomolecule partitioning between two immiscible aqueous phases without chaotropic reagents or specialized consumables, providing a fast, affordable, and easily scalable method that requires only simple centrifugation for phase separation^25^.

In our previous work^26^, we demonstrated efficient and robust capture of short DNA fragments from plasma using polyethylene glycol (PEG)/phosphate ATPS and introduced a solid-component design to increase plasma input while minimizing dilution. Coupling the extraction with a reverse elution step further improved DNA concentration and purity. However, the resulting concentrations remained below the input requirements of standard NGS library preparation kits, and the influence of a lysis step had not yet been investigated.

Building on this foundation, the present study optimizes the ATPS-based extraction process to address these limitations. We assess the effects of different lysis conditions, identify alternative system points for higher DNA concentration with minimal loss, and explore additional strategies, such as serial ATPS, for further enrichment. The optimized workflow is evaluated for PCR inhibition and compared to the QIAamp Circulating Nucleic Acid Kit (QIAGEN) using varying DNA inputs and fragment lengths. Finally, the complete ATPS-based process is applied to a spiked cfDNA reference set for NGS analysis, demonstrating that the method can yield sequencing-quality DNA suitable for downstream molecular analyses.

## Results

### System optimization

In our previous work^26^, a promising ATPS composition (19.5% (w/w) PEG/13.9% (w/w) salt; tie-line length 41%) was identified, which enabled efficient DNA capture in the bottom phase while separating it from the main plasma protein fraction. However, the DNA concentration in the final extract remained too low for reliable downstream analysis. Here, DNA concentration is expressed as a concentration factor, defined as the ratio of the DNA concentration in the final extract to that in the plasma input.

#### Effect of tie-line length and phase volume ratio

To increase DNA concentration without altering phase distribution, system compositions along different tie-lines were investigated. Previously, increasing PEG concentration to reduce bottom-phase volume led to DNA loss. To prevent this while maintaining a high phase volume ratio ($V_{R}$), a system point on a shorter tie-line, requiring lower PEG and salt concentrations to induce phase separation, was tested. Preliminary screening experiments identified a composition with a theoretical $V_{R}$ of five and tie-line length of 34% (27.1% (w/w) PEG/8.1% (w/w) salt), which doubled the DNA concentration in the bottom phase without affecting recovery (70%) (Fig. S1a). Protein distribution remained comparable to the reference system, confirming that selectivity was maintained.

#### DNA concentration by reverse elution

**Fig. 1:**
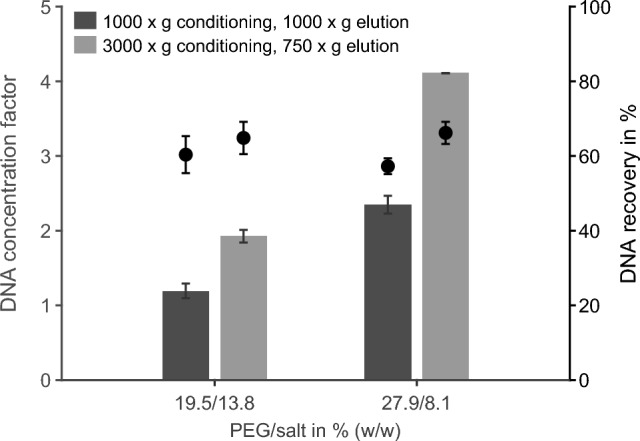
Effect of system composition and purification protocol on DNA recovery and concentration factor. DNA concentration factor (bars, left axis) and recovery (dots, right axis) are shown for two ATPS compositions with different PEG/salt contents (% (w/w)). Bottom-phase samples were purified using either the optimized (3000 $\times$ g conditioning, 750 $\times$ g elution) or the standard reverse elution protocol (1000 $\times$ g for both steps). Data represent mean ± SD of biological duplicates, each measured in technical triplicates.

To further enhance DNA concentration, the optimized system point was combined with the previously described reverse elution protocol^26^. Figure 1 shows DNA recoveries and concentration factors obtained with the standard and concentration purification protocols. The optimized ATPS composition with a higher PEG content doubled the DNA concentration factor relative to the reference system, without affecting recovery significantly (57%-66%). Applying the optimized reverse elution protocol (3000 $\times$ g conditioning, 750 $\times$ g elution) nearly doubled the factor again (from 2.3 to 4.1), yielding an overall fourfold increase compared to the initial system. Moreover, conductivity measurements confirmed efficient salt removal for all conditions (Fig. S1b). Combining the optimized ATPS composition with the adapted reverse elution protocol substantially improved DNA concentration without compromising recovery or protein removal efficiency.

### Effect of lysis on extraction efficiency

**Figure 2:**
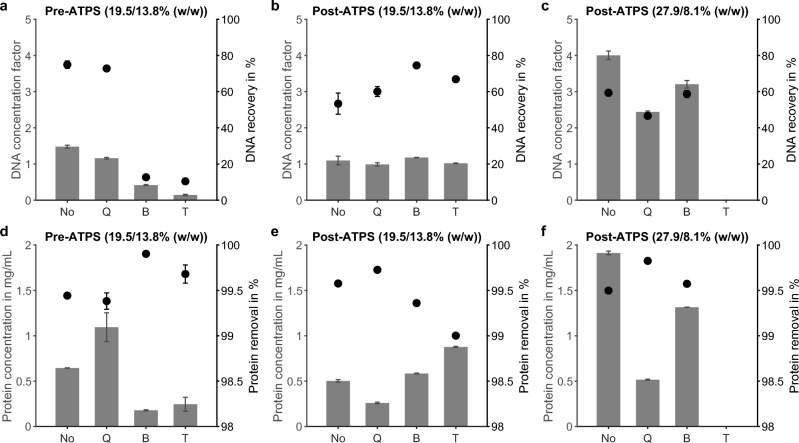
Impact of pre- and post-ATPS capture lysis methods on DNA and protein recovery in the bottom phase. (**a**–**c**) DNA concentration factor (bars, left axis) and recovery (dots, right axis) for two ATPS compositions with different PEG/salt contents. Lysis was performed on plasma pre-capture (**a**) or on the bottom phase post-capture (**b**,**c**) using enzymatic (Q), buffer-based (B), or reducing (T) methods, compared to a non-lysed reference (No). (**d**–**f**) Corresponding protein concentration (bars, left axis) and removal efficiency (dots, right axis) under identical conditions. Data represent mean ± SD of technical triplicates.

To assess the effects of a lysis step in the ATPS-based extraction workflow, three different lysis methods were evaluated and compared with a non-lysed reference (No). The tested methods were Proteinase K digestion with QIAGEN lysis buffer ACL (Q), BioEcho lysis buffer with clearing solution (B), and lysis solution containing a reducing agent (T). For comparability, all systems contained identical concentrations of phase-forming components and were purified using the optimized reverse elution protocol. Figure 2 shows the resulting DNA concentration factors and recoveries, as well as protein concentrations and removal efficiency for the tested lysis conditions pre- and post-ATPS capture.

#### Plasma lysis pre-ATPS capture

When plasma was lysed before ATPS formation, phase behavior differed from the non-lysed reference, showing a thinner or more flocculent interphase depending on the lysis method (Fig. S2a). Despite these visual differences, protein removal remained high (99.4%–99.9%) across all conditions. DNA recovery was highest in the non-lysed system (75%±2%) and comparable for lysis Q, while recoveries for lysis B and T were below 13%.

#### Bottom phase lysis post-ATPS capture

Applying lysis to the DNA-containing bottom phase after capture with the reference ATPS (19.5% (w/w) PEG/13.9% (w/w) salt) yielded comparable DNA concentration factors (1.0-1.2) across all methods. After accounting for dilution, recoveries reached approximately 75% for lysis B, 60% for lysis Q, and 53%±6% for the non-lysed reference. Protein removal again exceeded 99.0% for all methods, but SDS-PAGE analysis revealed differences in residual protein bands (Fig. S2b). In the non-lysed bottom phase, distinct bands were observed at 66 kDa (human serum albumin (HSA)), 55 kDa (transferrin), $\sim$45 kDa ($\alpha$1-antitrypsin), and 23 kDa (apolipoprotein A1). These bands appeared fainter after purification, confirming protein removal. Following lysis, the intensity and number of visible bands decreased: lysis T showed bands near 55 kDa, 45 kDa and 23 kDa, lysis B retained the 23 kDa band and a faint one around 45 kDa, and lysis Q displayed the $\sim$45 kDa band as well as a smear around 6 kDa. Conductivity values were slightly lower for lysed samples (0.5 mS/cm) than for the non-lysed bottom phase (0.9 mS/cm), suggesting minor salt precipitation effects (Fig. S2c).

For the optimized ATPS composition (27.1% (w/w) PEG/8.1% (w/w) salt), protein removal efficiency remained high (99.5%), and lysis showed no considerable benefit. The non-lysed sample achieved the highest DNA recovery (59%) and concentration factor (4.0). Lysis B produced a similar recovery but a lower concentration factor of 3.2 due to dilution with lysis buffer, while lysis Q performed less effectively with a recovery of 47%.

Overall, the reduction-based lysis method B achieved recoveries comparable to or higher than those of the enzymatic method Q, despite its shorter incubation time (2 min vs. 30 min) and the absence of enzymes. However, depending on the system composition, omitting lysis completely yielded equally efficient, if not higher, DNA extraction.

### Performance comparison with silica-based extraction

**Figure 3:**
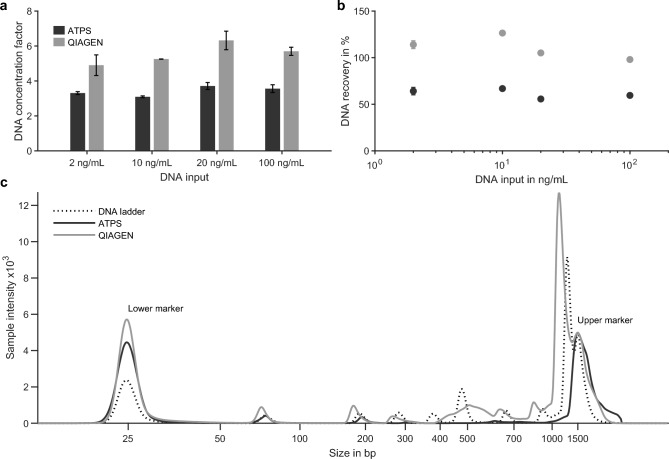
Comparative performance of ATPS- and adsorption-based QIAGEN extraction. (**a**) DNA concentration factor as a function of input concentration (2–100 ng/mL) for ATPS and QIAGEN extractions. (**b**) DNA recovery as a function of input concentration. (**c**) Fragment size distribution of a 1 kbp DNA ladder before and after extraction with both methods, showing relative fluorescence intensity over fragment length (bp). Input analyses were performed in technical triplicate. Fragment-length analysis was performed on a representative sample.

The performance of the optimized, lysis-free ATPS-based extraction and purification process was evaluated and compared to the QIAamp Circulating Nucleic Acid kit (QIAGEN) across a range of DNA input concentrations and fragment lengths. Fig. 3 shows the resulting DNA concentration factors and recoveries for a 160 bp DNA fragment as well as the fragment size distribution of a 1 kb DNA ladder after extraction.

#### Effect of DNA input concentration

Plasma samples were spiked with a 160 bp DNA fragment at concentrations from 2 ng/mL to 100 ng/mL and extracted in parallel using the ATPS-based process and the QIAamp kit (QIAGEN). Due to elution in a small final volume (<100 $\upmu$L), the QIAamp kit achieved higher concentration factors (4.9-6.3) across the tested input range. Nevertheless, the ATPS process yielded consistent recoveries above 60% even at low DNA inputs (e.g., 1 ng DNA in 500 $\upmu$L plasma), indicating robust extraction efficiency. The recoveries calculated for QIAamp exceeded 100%, likely due to differences in the blank/reference (water) and sample (elution buffer) compositions. Reported recoveries for this kit typically range between 70–80%^27^. While the QIAamp kit achieved higher apparent recoveries, its workflow was approximately six times longer ($\sim$60 min) due to multiple incubation, vacuum, and centrifugation steps (Fig. S6). In contrast, the ATPS-based process required only a mixing step and centrifugation for phase separation and purification, offering reduced processing time ($\sim$10 min turnaround, $\sim$2 min hands-on) and straightforward scalability, particularly for larger plasma volumes.

#### DNA fragment size distribution and integrity

Microelectrophoresis confirmed that the spiked 160 bp PCR fragment was successfully recovered using both extraction methods (Fig. S3a,b). The analysis also showed that desalting is essential for accurate fragment detection, as DNA bands and size markers did not migrate correctly in unpurified samples. To evaluate DNA integrity and size distribution, additional experiments were performed by spiking plasma with a 1 kb DNA ladder. The ATPS-based extraction efficiently recovered fragments within the cfDNA size range, while fragments larger than 750 bp were largely absent from the extract. In contrast, the QIAamp extraction yielded higher overall DNA concentration for all fragment sizes but displayed broader or poorly resolved peaks for fragments above 270 bp, suggesting possible co-extraction of aggregated or high-molecular-weight DNA.

To further confirm the size selectivity of ATPS, plasma spiked with lambda DNA (48.5 kbp) was analyzed. More than 70% of the lambda DNA was recovered in the interphase, while none was detected in the bottom phase, confirming effective exclusion of long DNA fragments such as contaminating gDNA. The thicker interphase observed for lambda DNA samples visually corroborated these findings (Fig. S3c,d).

ALU-quantitative polymerase chain reaction (qPCR) was performed on extracts from unspiked, healthy plasma to detect endogenous cfDNA and determine DNA integrity index (DI) following the method of Umetani et al.^28^. The DI of the QIAamp extract was 0.5, consistent with the presence of DNA fragments longer than 247 bp. In contrast, DI values for the ATPS extracts ranged from 0.1 to 0.2, indicating that nearly all DNA was truncated into fragments smaller than 247 bp. These results align with the electrophoresis data, confirming the size-selective recovery of short cfDNA-like fragments in ATPS.

### Assessment of qPCR compatibility

**Figure 4:**
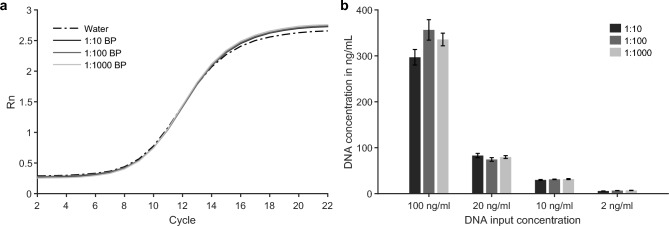
Quantitative PCR performance for spike-in experiments and dilution series to assess matrix inhibition. (a) Amplification curves for 160 bp DNA spiked at 5 ng/mL into water and into a dilution series of the ATPS bottom phase extracts (BP) to evaluate potential matrix inhibition of PCR amplification. Fluorescence intensity is plotted against cycle number. Each condition was measured in technical triplicate; one representative amplification curve per condition is shown for clarity. (b) Quantified DNA concentrations for input concentrations of 2-100 ng/mL measured in three dilutions (1:10, 1:100, 1:1000). Data represent mean ± SD of technical triplicates.

To assess potential inhibition of amplification-based techniques by matrix components in the purified ATPS extracts, spike-in experiments were evaluated using qPCR. Fig. 4a shows the amplification curves for a dilution series of blank extracts spiked with 5 ng/mL DNA, displayed for one representative replicate per condition for clarity (all curves shown in Fig. S4a). The curves exhibit the expected sigmoidal shape with minimal variation between triplicates, indicating the absence of inhibitory effects. The quantified DNA concentrations (4.9-5.5 ng/mL) closely matched the expected values, further confirming the lack of inhibition. A qualitative comparison with QIAamp extracts likewise showed comparable amplification performance, indicating that the ATPS workflow does not introduce detectable inhibitors unique to the extraction (Fig. S4b). Differences in Ct values between ATPS and QIAamp extracts corresponded to the respective DNA concentrations obtained after extraction. Similarly, lysed samples showed efficient amplification, suggesting that purification via reverse elution effectively removed potentially interfering components from the lysis step. Consistent amplification behavior across diluted samples from the DNA input experiments further supports the high purity and qPCR compatibility of ATPS extracts (Fig. 4b).

### Serial ATPS extraction for enhanced DNA concentration

To further increase DNA concentration, the efficiency of serial ATPS steps was evaluated. In this setup, fresh PEG was added to the bottom phase of a first ATPS to induce a second phase separation, aiming to reduce the bottom-phase volume and remove residual protein. Due to mass balance constraints, adding PEG alone did not reproduce the composition of the first ATPS, so fresh plasma was added to dilute the rest of the phase-forming components. Although this increased total volume, it enabled reaching the desired PEG/salt content while introducing additional DNA into the system. This is a beneficial feature for liquid biopsy applications where larger plasma volumes increase the chance of recovering rare cfDNA fragments^29^.

DNA recovery and concentration factors were analyzed across three consecutive ATPS steps (Table 1). Samples were diluted tenfold before analysis but were not purified by reverse elution due to sample volume constraints, each bottom phase being used to create the subsequent ATPS. Recovery for each step was calculated relative to the total DNA present before phase separation, including DNA added through fresh plasma and carried over from the preceding step. The total recovery was based on the total DNA input across all steps.

**Table 1:** Parameters and analytical outcomes for serial ATPS steps. Overview of system parameters and analytical results for consecutive ATPS steps, including plasma input, bottom phase (BP) volume, concentration factors (CF), DNA recovery, protein concentration, and protein removal efficiency before reverse elution. Data is reported for a representative experiment.

ATPS	Plasma input	BP volume	BP CF	DNA CF	DNA recovery	Protein concentration	Protein removal
1	1000 $\upmu$L	215 $\upmu$L	4.7	3.3±0.4	71±8%	3.0 mg/mL	99.0%
2	229 $\upmu$L	145 $\upmu$L	3.1	4.5±0.3	70±5%	1.5 mg/mL	98.6%
3	154 $\upmu$L	103 $\upmu$L	2.9	6.2±0.3	79±4%	1.3 mg/mL	98.7%
Total	1383 $\upmu$L	103 $\upmu$L	13.4	6.2±0.3	46±3%	1.3 mg/mL	99.8%

Serial ATPS formation was successfully achieved, though bottom-phase volume reduction for systems 2 and 3 was limited to a factor of 3, compared to 4.7 for system 1. Total protein removal reached 99.8% before reverse elution, confirming efficient protein depletion across all steps. Individual step recoveries were consistent (70-79%), yet cumulative recovery decreased to 53±4% after the second step and 46±3% after the third. Thus, while serial extraction increases plasma input and DNA concentration, it does so at the expense of overall yield. This trade-off requires careful optimization.

### Process optimization

#### Selection of optimal system composition

**Fig. 5:**
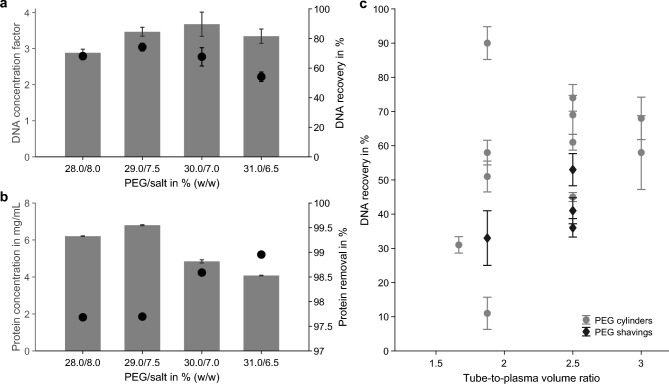
Effects of system composition and tube-to-plasma volume ratio on DNA recovery. (**a**) DNA concentration factor (bars, left axis) and DNA recovery (dots, right axis) before reverse elution for ATPS compositions with different PEG/salt contents (% (w/w)). (**b**) Corresponding protein concentration (bars, left axis) and removal efficiency (dots, right axis) under the same conditions. (**c**) DNA recovery of unpurified bottom phase samples as a function of tube-to-plasma volume ratio for PEG cylinders (dots) and PEG shavings (diamonds). Data represent mean ± SD of technical triplicates.

Following these findings, a second screening was conducted to identify the optimal system-point composition that maximized bottom-phase volume reduction without compromising DNA recovery. Based on previously established phase diagrams^30^, four system points with increasing PEG and decreasing salt concentrations were tested. The first composition was chosen to be similar to the previously optimized composition (27.9% (w/w) PEG/8.1% (w/w) salt). The samples were diluted tenfold prior to analysis, but were not purified by reverse elution. As expected, the plasma input to bottom-phase volume ratio increased from 4.2 to 6.2 with higher PEG content, but DNA recovery declined by more than 10% at a system composition of 31.0/6.5% (w/w) (Fig. 5a). Interestingly, protein concentration in the bottom phase decreased at PEG concentrations above 30% (w/w), indicating improved protein removal (Fig. 5b). The ATPS with 30.0% (w/w) PEG and 7.0% (w/w) salt provided the best compromise between DNA concentration, recovery, protein removal, and volume reduction, and was selected for subsequent experiments.

#### Influence of practical handling parameters

To streamline the ATPS-based extraction process, several practical parameters were evaluated, including PEG dissolution, tube filling ratio, component addition order, incubation temperature, and centrifugation time.

To accelerate PEG dissolution, thin shavings obtained from solidified PEG films were tested instead of cylindrical PEG pieces. Although initial dissolution appeared faster, total dissolution time was not reduced, and DNA recovery decreased to 36–53% compared to 45–74% with cylindrical PEG for the same tube-to-plasma volume ratio of 2.5 (Fig. 5c).

Process performance was also affected by the total ATPS volume relative to tube volume. Systems with a tube-to-plasma volume ratio below two showed large variability in DNA recovery (10–90%), likely due to insufficient mixing and incomplete dissolution of PEG and salt. A ratio of at least two is therefore recommended to ensure reproducible results.

The order of component addition had a minor but reproducible influence. Optimal performance was achieved when plasma was first mixed with the salt before adding PEG, using cylindrical pieces smaller than 150 mg rather than shavings.

Incubation temperature during mixing was evaluated by increasing it between $30^{\circ }\hbox {C}$ and $40^{\circ }\hbox {C}$ compared to room temperature. Contrary to expectations based on PEG 1000’s melting point ($35\text {-}40^{\circ }\hbox {C}$), higher temperature did not accelerate dissolution, and led to increased protein partitioning into the bottom phase, indicating reduced selectivity (Fig. S5a). Room-temperature incubation was therefore selected for the final protocol, which also simplifies processing by eliminating the need for heating during mixing.

Finally, centrifugation time for phase separation was reduced from 5 min to 1 min without affecting phase formation (Fig. S5b), DNA recovery, or protein distribution. The resulting optimized workflow is summarized in Fig. S6a.

#### Optimization of reverse elution conditions

Lastly, modifications to the reverse elution protocol were tested to improve DNA recovery and concentration. Specifically, it was observed that reusing purification plates that had undergone repeated high-speed centrifugation (3000 $\times$ g) led to substantial DNA loss, suggesting that DNA may become trapped within the damaged matrix. To verify this, the eluate was reapplied to the same well and eluted again at 750 $\times$ g for 1 min. The second elution yielded the same final volume as the first, but approximately twice the DNA amount was recovered, confirming that a large portion of DNA remained in the matrix after the first elution. While this double-elution step can effectively recover retained DNA, it also increases the risk of co-eluting residual proteins or salts. Therefore, using fresh plates that have not been centrifuged above 1000 $\times$ g more than once is recommended to minimize DNA retention.

**Fig. 6:**
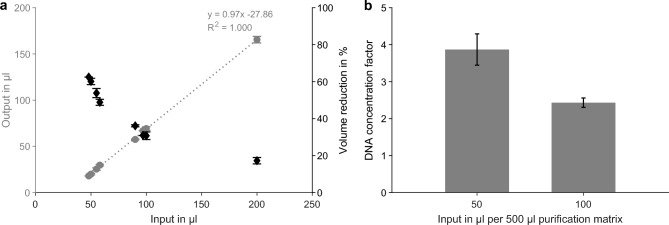
Correlation between input and output volumes during reverse elution and their effect on DNA concentration. (a) Output volume (dots, left axis) and relative volume reduction (diamonds, right axis) as a function of input volume during reverse elution using the optimized protocol (3000 $\times$ g conditioning, 750 $\times$ g elution), including a linear trend line. (b) DNA concentration factor for 100 $\upmu$L and 50 $\upmu$L input extract per well containing 500 $\upmu$L purification matrix. Data in (a) represent mean ± SD from at least two independent experiments for each input volume (n $\ge$ 12 for 50 $\upmu$L and 100 $\upmu$L input). Data in (b) represent mean ± SD of technical triplicates.

Reducing the input volume per well proved more directly beneficial for DNA concentration. In the standard protocol, 100 $\upmu$L of the bottom phase were applied per well, containing 500 $\upmu$L of the purification matrix. Experiments showed that the retained volume after conditioning at 3000 $\times$ g and elution at 750 $\times$ g remained approximately 30 $\upmu$L, regardless of the input volume. Consequently, decreasing the input from 100 $\upmu$L to 50 $\upmu$L increased the relative volume reduction from 30.8% to 60.1% (Fig. 6a). When 100 $\upmu$L of bottom phase were split into two wells (50 $\upmu$L each, eluates pooled), the DNA concentration factor increased from 2.4 to 3.9 without affecting recovery (Fig. 6b). These results demonstrate that DNA concentration can be improved by lowering the input volume per well while maintaining recovery and desalting efficiency.

### Extraction of cfDNA reference material for sequencing analysis

As a proof-of-concept demonstration of sequencing compatibility, a cfDNA reference set containing defined variants was spiked into DNA-free plasma and processed using the developed ATPS-based extraction workflow. Sequencing analysis performed in a routine molecular pathology laboratory at a university clinic demonstrated that the resulting extracts enabled reliable NGS library preparation, with expected quality-control metrics, including TapeStation profiles and library concentrations. In addition, on-target deduplication ratio and median fragment lengths obtained on a NovaSeq 6000 sequencer (Illumina) were also within the expected range (Tab. S2). We further compared a single-step ATPS extraction with a serial two-step ATPS process. In both cases, all variants spiked at 5% variant allele frequency (VAF) were reliably detected, whereas no variants were observed in the negative controls spiked with 0% VAF cfDNA (Tab. 2). These results demonstrate that DNA obtained via the ATPS extraction workflow is compatible with downstream library preparation and NGS-based variant detection.

**Table 2:** Next-generation sequencing results for cfDNA reference material with defined mutations extracted using the ATPS-based process. DNA-free plasma was spiked at 5% variant allele frequency (VAF). Detected variants, total sequencing depth, and measured VAF are reported for extracts obtained using a single ATPS step (ATPS 1) or two consecutive ATPS steps (ATPS 2). Data is reported for a representative experiment.

Variant			ATPS 1		ATPS 2	
Gene	cDNA	Protein	Depth	VAF	Depth	VAF
AKT1	c.49G>A	p.E17K	4702	0.070	6789	0.068
BRAF	c.1799T>A	p.V600E	3431	0.055	4851	0.059
ERBB2	c.2313_2324dup	p.Y772_A775dup	7473	0.059	10915	0.060
KRAS	c.35G>A	p.G12D	4433	0.067	6471	0.075
KRAS	c.181C>A	p.Q61K	5151	0.086	7569	0.088
KRAS	c.436G>A	p.A146T	2964	0.054	4324	0.045
PIK3CA	c.1633G>A	p.E545K	2885	0.063	4169	0.052
PIK3CA	c.3140A>G	p.H1047R	4320	0.104	6488	0.102

## Discussion

In this study, we developed and optimized an ATPS-based workflow to extract and purify short DNA fragments from plasma, demonstrating its potential for liquid biopsy applications. Although DNA recovery has not yet reached the efficiency of state-of-the-art adsorption-based methods such as the QIAamp kit (QIAGEN)^21^, the presented method offers a rapid, simple, and cost-effective alternative that requires minimal equipment and handling. Furthermore, the ATPS-based workflow enabled reproducible DNA recovery from minute input amounts, efficient protein removal, compatibility with amplification-based downstream analyses, and robust performance under optimized parameters. This approach could facilitate the clinical implementation of cfDNA-based liquid biopsy assays, advancing individualized disease monitoring and treatment^5^.

DNA concentration is critical for NGS library preparation, as most kits require defined input amounts of DNA in small volumes (e.g., 10 ng in 50 $\upmu$L), and cfDNA concentrations in plasma typically range from 1 to 40 ng/mL^10^. The initially selected ATPS composition provided high recovery, but limited volume reduction ($V_{R}$=1). Shifting along the same tie-line to increase $V_{R}$ caused substantial DNA loss^26^, likely due to PEG-induced DNA precipitation^31,32^. A shorter tie-line was identified where DNA and protein partitioning remained consistent, yet phase formation required less PEG and salt, enabling an effective reduction in bottom-phase volume while maintaining $\sim$60% recovery after purification. These findings confirm that ATPS composition can be tuned to balance recovery and selectivity, though approaching the binodal too closely reduces robustness and alters partitioning behavior^33^, as observed before^26^. The second system optimization highlights the influence of PEG exclusion effects and solubility limits on DNA recovery^34,35^, as well as the inherent trade-off between concentration and yield in ATPS-based extraction.

Serial extraction experiments, inspired by back-extraction approaches found in the literature^36,37^, demonstrated that repeated ATPS steps can effectively increase DNA concentration and total plasma input. However, each step introduced incremental DNA loss, ultimately reducing total yield to about half after three ATPS. Despite the consistent formation of biphasic systems and enhanced protein removal, deviations in phase volume reduction in later cycles suggest compositional shifts. While PEG and salt ratios were theoretically identical across all systems, the lower plasma and protein content in systems 2 and 3 likely influenced phase formation and shifted the binodal, as previously observed for biomass-dependent phase behavior^38^. Thus, while serial extraction can improve concentration and purity, its advantages must be weighed against the cumulative DNA losses it entails.

The influence of lysis on extraction performance was evaluated both pre- and post-ATPS capture to determine whether additional disruption of protein-DNA complexes could enhance recovery. The results indicate that plasma lysis prior to ATPS formation is not beneficial, as it disrupts phase behavior due to altered protein composition and plasma dilution. Because each lysis method relies on different mechanisms (enzymatic digestion, reduction, precipitation), the resulting interphase appearance and composition vary accordingly. This was confirmed by SDS-PAGE analysis of lysed bottom-phase samples, which revealed method-specific protein band patterns reflecting distinct degradation pathways. Proteinase K is a serine protease that cleaves peptide bonds^39^ and produces a broad smear of low-molecular-weight fragments, whereas the strong reduction agent used in lysis T disrupts disulfide bonds, yielding smaller protein subunits^40^. However, without a precipitating agent such as the clearing solution in lysis B, these subunits remain in solution.

In contrast, applying lysis post-ATPS, when most proteins have already been removed from the bottom phase, increased DNA recovery by up to 20%. This suggests that disrupting residual DNA-protein interactions within the bottom phase facilitates DNA release during purification. However, the benefit of post-capture lysis was not consistent across all systems. In the ATPS containing 7.6% (w/w) more PEG and 5.7% (w/w) less salt, the non-lysed sample performed best. This likely reflects unfavorable interactions between lysis reagents and phase-forming components, most likely the high amount of PEG, which could lead to increased PEG–salt precipitation^41^.

Among the tested lysis methods, the rapid, enzyme-free lysis B achieved comparable recovery to the enzymatic standard while drastically reducing incubation time. Method Q was optimized for proprietary buffer systems and plasma lysis to increase DNA adsorption onto silica, which explains its reduced effectiveness in lysing high-salt bottom-phase samples. The results highlight that post-capture lysis can improve DNA yield, but is not strictly required, as the salt-rich bottom phase itself likely promotes histone dissociation and cfDNA release^42^. Because cfDNA is naturally associated with histones, conventional extraction workflows typically rely on lysis using strong chaotropes such as guanidine thiocyanate to improve DNA adsorption to silica membranes or beads^43,44^. In ATPS systems, omitting this step could streamline the workflow, reduce reagent consumption, and facilitate automation, supporting potential clinical implementation.

Process parameter optimization revealed that seemingly minor handling conditions can impact the reproducibility and efficiency of ATPS-based DNA extraction. Attempts to accelerate PEG dissolution by using thin shavings unexpectedly reduced DNA recovery, likely due to transient local PEG concentration peaks that promote DNA precipitation, as previously observed for pre-dissolved PEG systems^26^. In contrast, slower, more gradual dissolution of larger PEG pieces improved DNA recovery, highlighting the importance of homogeneous component mixing and complete equilibration before phase separation^45,46^. Similarly, systems processed in overfilled tubes (tube-to-plasma ratios <2) showed high variability, indicating insufficient mixing and impaired mass transfer. These conditions could lead to increased DNA entrapment in the interphase rather than transfer to the protein-poor bottom phase. This hypothesis is supported by recent studies showing that mixing time and agitation speed influence partitioning and mass transfer in ATPS^47^. In addition, vessel geometry, particularly the height-to-diameter ratio, has been shown to affect phase separation kinetics and equilibrium in ATPS, further emphasizing the role of physical process parameters in system performance^48^.

Further tests showed that elevated temperatures during mixing did not accelerate PEG dissolution but instead led to undesired protein partitioning into the bottom phase. Such temperature-induced binodal shifts have been reported previously for PEG-salt ATPS^49^, and elevated temperature can also alter protein stability. Thus, room-temperature operation preserves selectivity while simplifying handling. Although the equilibration time could not be shortened without compromising performance, the centrifugation time was reduced from 5 min to 1 min without affecting phase formation or analyte distribution, thereby improving process throughput. Together, these findings demonstrate that control of practical handling parameters such as mixing, temperature, and tube volume is essential for reliable ATPS performance. Maintaining a simple, room-temperature workflow supports scalability and facilitates integration into automated extraction platforms. High-throughput processing could be achieved using a deep-well plate format and a liquid-handling station equipped with an integrated centrifuge and a rotational shaker, as in the setup described in our previous work^30^.

Purification by reverse elution was optimized to increase DNA concentration and purity. As shown previously for 160 bp DNA fragments in water^26^, higher conditioning speeds effectively “dried” the purification matrix, thereby allowing greater retention of the input liquid within pores and interparticle spaces^50^. Combined with a lower elution rate, which prolongs liquid-matrix contact, this enabled efficient flow-through volume reduction. Although DNA loss was slightly higher for real bottom-phase samples than for spiked water, the purification step remains essential for removing excess salts and residual proteins that can interfere with downstream analyses. DNA retention in the matrix likely arises from entrapment within interstitial spaces by capillary forces^51^, weak electrostatic interactions^52^, or, in the case of plasma extracts, associations with salt and proteins.

When purification plates previously exposed to high-speed centrifugation were reused, DNA retention increased substantially. This suggests that mechanical stress during conditioning damages purification matrix particles, altering pore structure and binding capacity^53,54^. While a second elution can recover most of the retained DNA, it also risks co-eluting residual contaminants, making the use of fresh plates preferable. In contrast, reducing the input-to-matrix ratio improved concentration performance without compromising recovery or purity, thereby providing a simple means to increase the final DNA concentration factor. Further tuning of this ratio could enhance performance, though too little matrix risks contaminant breakthrough, while excess matrix increases DNA retention. Overall, these insights demonstrate that physical handling parameters, rather than matrix chemistry alone, can substantially influence the final yield and concentration in the purification step.

The analytical performance of the ATPS extracts confirmed their suitability for amplification-based downstream analysis. qPCR spike-in experiments inspired by Devonshire et al.^14^ showed no evidence of inhibition^55^, and measured concentrations closely matched expected values, indicating efficient removal of potentially interfering plasma and phase-forming components. ALU-qPCR analysis of unspiked plasma further demonstrated that ATPS extracts contained predominantly short DNA fragments (<247 bp)^28^, whereas silica-based extraction also retained longer fragments. The resulting low DNA integrity index aligns with the characteristic size distribution of ctDNA in plasma^12^, and electropherograms confirmed the efficient exclusion of longer fragments from the bottom phase. This selective recovery reduces gDNA contamination while efficiently retaining cfDNA^13^. The preferential recovery of shorter fragments suggests that the ATPS extraction system introduces a degree of size selectivity. Such enrichment may be advantageous for mutation-based liquid biopsy assays, as tumor-derived DNA fragments are typically shorter than background cfDNA^56–58^. Similar size-selective approaches have been reported to increase the relative abundance of tumor-derived variants^37,59^. For applications requiring fully unbiased genome-wide fragment representation, the potential influence of extraction-related size selection should be evaluated in future studies.

To assess the compatibility of ATPS-derived extracts with sequencing workflows, proof-of-concept NGS experiments were performed in a clinical molecular pathology laboratory with experience in liquid biopsy diagnostics for solid tumors. Library preparation was consistently successful for all ATPS extracts, indicating the absence of inhibitory residues and sufficient cfDNA quality. Sequencing quality metrics were within expected ranges, enabling reliable variant calling at adequate depth. According to the sequencing laboratory, the library preparation and sequencing quality metrics obtained from ATPS-extracted samples were comparable to those typically observed for routine plasma samples processed in their laboratory using the Maxwell®RSC Rapid ccfDNA Kit (Promega), which had previously been internally benchmarked against a QIAamp-based extraction workflow (personal communication). Together, these results demonstrate that cfDNA purified using the developed ATPS-based workflow is compatible with downstream NGS analysis. However, this study does not include a direct, comprehensive comparison with established cfDNA extraction methods, and future studies should demonstrate that variant detection and library complexity are comparable.

Similar to the microfluidic PIBEX approach developed by Lee et al.^51^, the presented workflow addresses several practical limitations associated with conventional silica-based extraction methods, such as the manual QIAamp Circulating Nucleic Acid Kit, including the risk of cross-contamination during multi-step handling, limited input volumes, and relatively high consumable costs^19^. Based on comparative analyses reported in the literature, the recovery efficiency achieved with the ATPS-based method falls within the range reported for commonly used magnetic bead-based systems, such as the Maxwell RSC ccfDNA Plasma Kit (Promega,  60%) and the MagMAX Cell-Free DNA Isolation Kit (Applied Biosystems,  50%)^21,27^. Although further benchmarking against established workflows will be necessary, the simplicity of the phase-partitioning approach, its minimal handling requirements, and potential scalability suggest that the developed ATPS workflow represents a promising alternative to conventional adsorption-based cfDNA extraction strategies.

A limitation of the present study is that most experiments were performed using cfDNA reference material spiked into healthy plasma rather than patient-derived clinical samples. While this model system enabled controlled investigation of DNA partitioning behavior and extraction performance, clinical plasma samples may present additional complexities, including variable levels of genomic DNA contamination, nucleoprotein complexes, and heterogeneous cfDNA fragment length distributions^17,60^. Consequently, future validation using patient-derived plasma samples will be necessary to fully assess the robustness and clinical applicability of the ATPS-based extraction workflow.

In conclusion, this study demonstrates the feasibility of an ATPS-based workflow for the extraction and purification of cfDNA-like fragments from plasma under mild, aqueous conditions. The process operates without chemical or enzymatic lysis, enables efficient protein removal, and yields high DNA purity compatible with PCR and NGS analyses, all within a short processing time using minimal equipment. This makes it suitable for routine and potentially automated use. Although a trade-off between recovery and concentration remains, and validation with patient-derived plasma is still required, the presented method represents a considerable step toward more accessible cfDNA extraction. Building on prior work, this study addresses earlier limitations in concentration efficiency and lysis, establishing a proof of concept for an alternative ATPS-based extraction workflow for amplification-based liquid biopsy applications.

## Methods

### Blood collection and plasma processing

Blood samples were collected from healthy donors at the Institute for Experimental Hematology and Transfusion Medicine at the University Hospital Bonn (Bonn, Germany). The study procedures were performed in compliance with the approval granted by the Ethics Committee of the Medical Faculty at the University of Bonn (protocol code 070/05) and adhered to the Declaration of Helsinki. Written informed consent was obtained from all participants, and the procedures were carried out in accordance with institutional guidelines. After giving consent, approximately 10 mL of whole blood was collected into EDTA K3E S-Monovette collection tubes (Sarstedt, Nümbrecht, Germany) or Cell-Free DNA BCT^®^ (Streck, La Vista, NE, USA). Blood samples were shipped at room temperature and processed within 24 hours using a double centrifugation protocol to maximize plasma recovery. Whole blood was first centrifuged at 1600 $\times$ g for 10 min at room temperature. The plasma layer was then transferred and centrifuged at 16000 $\times$ g for 10 min to remove residual blood cells. Plasma aliquots of 1 mL were stored at $\hbox {-}80^{\circ }\hbox {C}$ until further use.

### Preparation of ATPS components

Starting points for system optimization were selected based on our previous work, where the experimental workflow and phase sampling procedure are described in detail^26^. To maximize the plasma input volume per ATPS, PEG 1000 (Merck KGaA, Darmstadt, Germany) and the phosphate salts $\hbox {NaH}_{2}\hbox {PO4}\cdot \hbox {H}_{2}\hbox {O}$ (Merck) and $\hbox {K}_{2}\hbox {HPO}_{4}$ (VWR International, Radnor, PA, USA) were added as solids rather than as pre-dissolved stock solutions. Cylindrical PEG pieces of up to 150 mg were prepared by melting the waxy polymer in a microwave and allowing it to solidify in a cylindrical mold for a few minutes. The salts were placed into Eppendorf tubes, mixed with spiked plasma, and finally combined with the PEG. Plasma aliquots were thawed at room temperature on the lab bench for 45 min, leaving any sedimented material undisturbed to avoid resuspension. Purified 160 bp DNA fragments were prepared as described by Meutelet et al.^30^ and gently mixed with plasma to the desired concentration before addition to the salts. For the NGS experiments, the 5-Gene-Multiplex Set cfDNA by SensID GmbH (Rostock, Germany) containing defined variants (AKT1/BRAF/ERBB2/KRAS/PIK3CA) at 0% and 5% VAF was spiked into human-tech DNA-free plasma (SensID GmbH). The amounts of phase-forming components and plasma varied with the investigated system point and experimental conditions. A summary of the relevant system points is provided in Table S1.

### ATPS-based DNA extraction protocol

A schematic overview of the developed extraction process is shown in Fig. S6a and comprises three main steps: ATPS capture, an optional second ATPS concentration step, and purification by reverse elution. Plasma was added to the tube containing phosphate salts, pulse-vortexed five times, followed by the addition of the PEG cylinders and another five pulse-vortexes. The tube was then mixed on a thermo-shaker at 1400 rpm and $25^{\circ }\hbox {C}$ for 5 min, followed by vortexing to ensure complete solubilization. Phase separation was achieved by centrifugation at 8000 $\times$ g for 1 min. The top phase was carefully removed without disturbing the interphase or bottom phase. The bottom phase was collected using a pipette by gently puncturing the interphase and transferring the liquid to a new tube. Any recoverable liquid adhering to the tube walls or trapped within the interphase was added to the bottom phase, and the final volume was measured manually with a pipette. The remaining interphase was discarded. For the optional serial ATPS step, fresh plasma and PEG 1000 were added to the recovered bottom phase. Mixing, centrifugation, and phase extraction were performed as described for the first ATPS.

The resulting salt-rich bottom phase was purified using reverse elution plates from the EchoCLEAN Organic Solvent DNA CleanUp Kit (BioEcho, Cologne, Germany). As described by Meutelet et al.^26^, plates containing 500 $\upmu$L matrix filling volume per well were centrifuged at 3000 $\times$ g for 1 min for conditioning, and elution was performed at 750 $\times$ g for 1 min. Each well was loaded with 100 $\upmu$L or 50 $\upmu$L, and the final purified DNA volume was measured manually using a pipette.

### Lysis conditions

Three lysis methods were evaluated and compared with a non-lysed sample: Proteinase K with lysis buffer from the QIAamp Circulating Nucleic Acid Kit (QIAGEN, Hilden, Germany) (Q), an enzyme-free lysis buffer with clearing solution (BioEcho) (B), and a reducing agent in water (T). Lysis was performed either on spiked plasma pre-ATPS capture or on bottom-phase samples before reverse elution. Reagent concentrations and volumes were adapted from the respective manufacturers’ protocols to match the plasma or bottom-phase volumes of the non-lysed sample.

For method Q, 100 $\upmu$L Proteinase K, 1 mL plasma, and 0.8 mL buffer ACL were combined, vortexed for 30 s, and incubated at $60^{\circ }\hbox {C}$ for 30 min. After cooling, the lysed plasma supernatant was used for ATPS formation. For methods B and T, 0.5 mL lysis buffer or 50 mM reducing agent solution was added to 1 mL plasma, incubated at $80^{\circ }\hbox {C}$ for 2 min, and cooled to room temperature. For lysis B, 43 $\upmu$L clearing solution was then added, and only the supernatant was used. The same procedures were applied to bottom-phase samples in post-capture lysis experiments. Conductivity of purified lysates was measured using an ÄKTApurifier conductivity sensor (Cytiva, Uppsala, Sweden).

### Reference adsorption-based extraction protocol

The adsorption-based QIAamp Circulating Nucleic Acid Kit (QIAGEN) was used as a reference extraction method. The protocol was performed according to the manufacturer’s instructions, except that carrier RNA was omitted. In brief, the workflow included sample lysis, DNA binding to silica columns, sequential washing with buffer and ethanol using a vacuum manifold, drying, and elution in water with a microcentrifuge. A schematic overview of the workflow, including detailed process parameters, is provided in Fig. S6b.

### Analytical methods

#### Protein quantification

Protein concentration in purified samples was measured in triplicate using a NanoDrop™ 2000/2000c UV/Vis spectrometer (Thermo Fisher Scientific). Water served as a blank, and tenfold-diluted plasma served as a reference for removal-efficiency calculations. An average extinction coefficient of 10 was assumed for plasma protein absorbance at 280 nm. Reducing SDS-PAGE analysis was performed on selected samples following the protocol and equipment described by Meutelet et al.^26^ to assess protein removal and composition after lysis and extraction.

#### DNA quantification

DNA was quantified using the Quant-iT™ PicoGreen™ dsDNA HS assay kit from Thermo Fisher Scientific Inc. (Waltham, MA, USA). Unpurified samples were diluted tenfold prior to analysis to reduce interference from phase-forming components. Analysis was performed as described by Meutelet et al.^26^, with blanks to account for background signal and references for recovery calculations. DNA recovery was calculated by multiplying the measured DNA concentration by the extract volume and dividing by the amount of input DNA.

#### Assessment of sample purity

qPCR was performed on selected purified samples to validate DNA quantification and assess sample purity after extraction, verifying the absence of amplification inhibitors. The assay targeted the spiked 160 bp DNA fragment. Reactions were performed in a total volume of 20 $\upmu$L, containing 5 $\upmu$L of sample or standard, 10 $\upmu$L of SsoAdvanced™ Universal SYBR^®^ Green Supermix (Bio-Rad Laboratories GmbH, Feldkirchen, Germany), and 0.25 $\upmu$M each of forward (CTACGGCAAGCTGACCCT) and reverse (GAAGCACTGCACGCCGTAG) primers (Sigma-Aldrich, St. Louis, MO, USA). All reactions were performed in triplicate on a QuantStudio™ 5 Real-Time PCR System (Thermo Fisher Scientific). Thermal cycling conditions comprised an initial denaturation at $95^{\circ }\hbox {C}$ for 7 min, followed by 40 cycles of denaturation at $95^{\circ }\hbox {C}$ for 15 s and annealing/extension at $60^{\circ }\hbox {C}$ for 20 s. Quantification was performed using a standard curve generated from serial dilutions of purified 160 bp DNA fragment (50 ng/mL to 0.005 ng/mL) and calculated by the Quant Studio™ Design & Analysis Software v1.5.1 (Thermo Fisher Scientific) based on cycle threshold (Ct) values. Amplification efficiency and linearity (R^2^) of the standard curve were verified for each run. No-template controls and blank extraction controls were included on each plate to confirm assay specificity and exclude contamination.

#### Evaluation of DNA integrity

DNA integrity was assessed using an ALU–based qPCR assay adapted from Umetani et al.^28^. Two primer sets targeting repetitive ALU sequences were used to amplify 115 bp and 247 bp fragments. Each 20 $\upmu$L reaction contained 10 $\upmu$L of SsoAdvanced™ Universal SYBR^®^ Green Supermix (Bio-Rad Laboratories GmbH), 0.15 $\upmu$M each of forward and reverse primer (either ALU115 or ALU247) (Sigma-Aldrich), and 5 $\upmu$L of sample or standard. Amplification was performed on the system described above with the following cycling conditions: initial denaturation at $95^{\circ }\hbox {C}$ for 10 min, followed by 35 cycles of $95^{\circ }\hbox {C}$ for 15 s and annealing at $64^{\circ }\hbox {C}$ for 1 min. DNA concentrations were determined from standard curves generated using serial dilutions of purified human genomic DNA (200-0.02 ng/mL), and the DNA integrity index (DI) was expressed as the ratio of ALU247 to ALU115 concentrations.

#### DNA fragment size analysis

DNA fragment size distribution was analyzed using the Agilent TapeStation 4150 system (Agilent Technologies, Santa Clara, CA, USA) with the D1000 ScreenTape assay, following the manufacturer’s instructions. Samples (1 $\upmu$L) were loaded together with the corresponding ladder, and electropherograms were evaluated using TapeStation Analysis Software. To investigate fragment size distribution in the extracts, the 160 bp PCR fragment was replaced with GeneRuler 1 kb Plus DNA ladder (Thermo Fisher Scientific).

#### Next-generation sequencing

NGS libraries for the customized LIQUIDPlex CSTM Solid Tumor panel (Archer, Inc./IDT, Boulder, CO, USA) were prepared according to the manufacturer’s instructions, using 35 ng cfDNA as input material and targeting 35 genes. After quantification with the NEBNext Library Quant Kit for Illumina (New England Biolabs, Ipswich, MA, USA) on a QuantStudio5 DX qPCR cycler (Thermo Fisher Scientific), libraries were paired-end sequenced for 300 cycles on a NovaSeq6000 sequencer (Illumina, San Diego, CA, USA). FASTQ files were generated on the Illumina DRAGEN v4 server and analyzed on the Archer analysis platform (version 7.3.1; Archer) with default settings.

#### Statistical analysis

Due to the exploratory nature of this proof-of-concept study and the limited availability of plasma samples, the number of biological replicates varied between experiments. Where possible, independent replicates were included to assess reproducibility. Measurements were generally performed in technical triplicate. Data are reported as mean ± standard deviation (SD). Error bars in figures represent SD of the indicated replicates. No formal statistical hypothesis testing was conducted, as the study was designed for exploratory validation rather than quantitative comparison.

## Supplementary Information

Supplementary Information.

## Data Availability

The datasets generated and analyzed during this study are available from the corresponding author upon reasonable request. No novel DNA or RNA sequences were generated in this work. Sequencing was performed exclusively for method validation using commercially available cfDNA reference material with predefined variants.
