# Supplementary material for: Proof of concept for aqueous two-phase system-based extraction of cell-free DNA from plasma for liquid biopsy applications
Source: Sci Rep. 2026 Apr 2;16:11232. doi: 10.1038/s41598-026-45585-z (PMC13046839; doi:10.1038/s41598-026-45585-z)
Supplement: Supplementary file 1 — Supplementary Information. [file 41598_2026_45585_MOESM1_ESM.pdf]

Proof of concept for aqueous two-phase system-based extraction of cell-free DNA from plasma for liquid biopsy applications

Rafaela Meutelet[1], Benedikt C Buerfent[2], Timo Hess[2,6], Julia Teply-Szymanski[3], Paul Jank[4], Johannes Oldenburg[5], Heiko Rühl[5], and Jürgen Hubbuch[1,*]

[1]Institute of Process Engineering in Life Sciences, Section IV: Biomolecular Separation Engineering, Karlsruhe Institute of Technology, Karlsruhe, 76131, Germany
[2]BioEcho Life Sciences GmbH, Cologne, 50829, Germany
[3]Institute of Pathology, Philipps-University Marburg and University Hospital Marburg (UKGM), Marburg, Germany
[4]Charité – Universitätsmedizin Berlin, corporate member of Freie Universität Berlin and Humboldt Universität zu Berlin, Department of Gynecology with Breast Center, Berlin, 10117, Germany
[5]Institute for Experimental Hematology and Transfusion Medicine, University Hospital Bonn, Bonn, 53127, Germany
[6]Institute of Human Genetics, University of Bonn, Bonn, Germany
[*]juergen.hubbuch@kit.edu

SUPPLEMENTARY INFORMATION

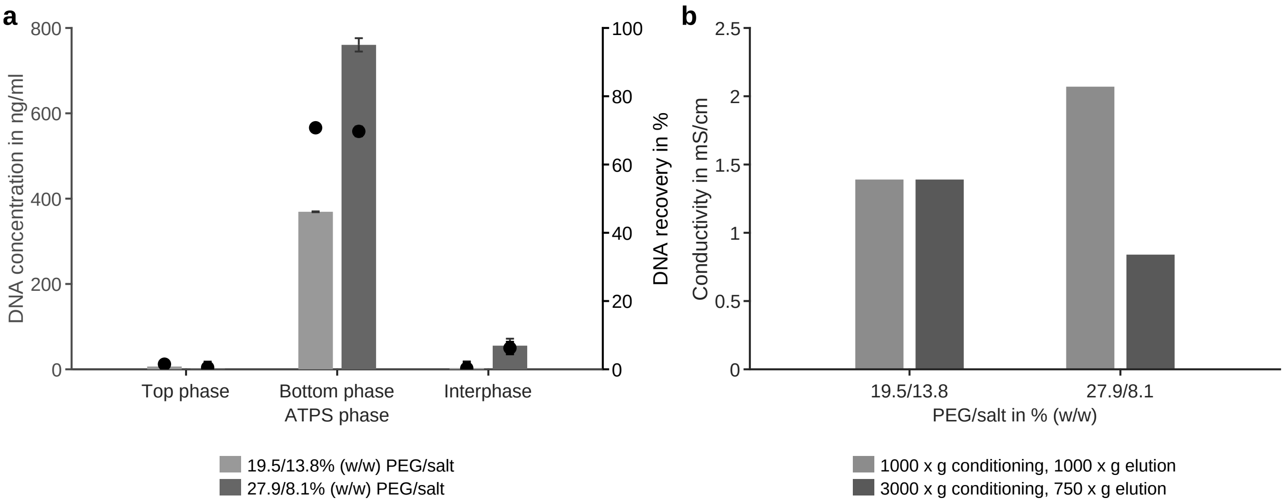

Figure S1. Effect of system composition and purification protocol on DNA partitioning and sample conductivity. (a) DNA concentration (left axis, bars) and recovery (right axis, dots) in the top phase, bottom phase, and interphase for two ATPS compositions with different PEG/salt ratios. Samples were purified using the standard reverse elution protocol (1000 × g for conditioning and elution). Data represent mean ± SD of technical triplicates. (b) Conductivity of bottom-phase samples from the two ATPS compositions after purification with either the standard or optimized reverse elution protocol (3000 × g conditioning, 750 × g elution). Measurements were performed once per sample.

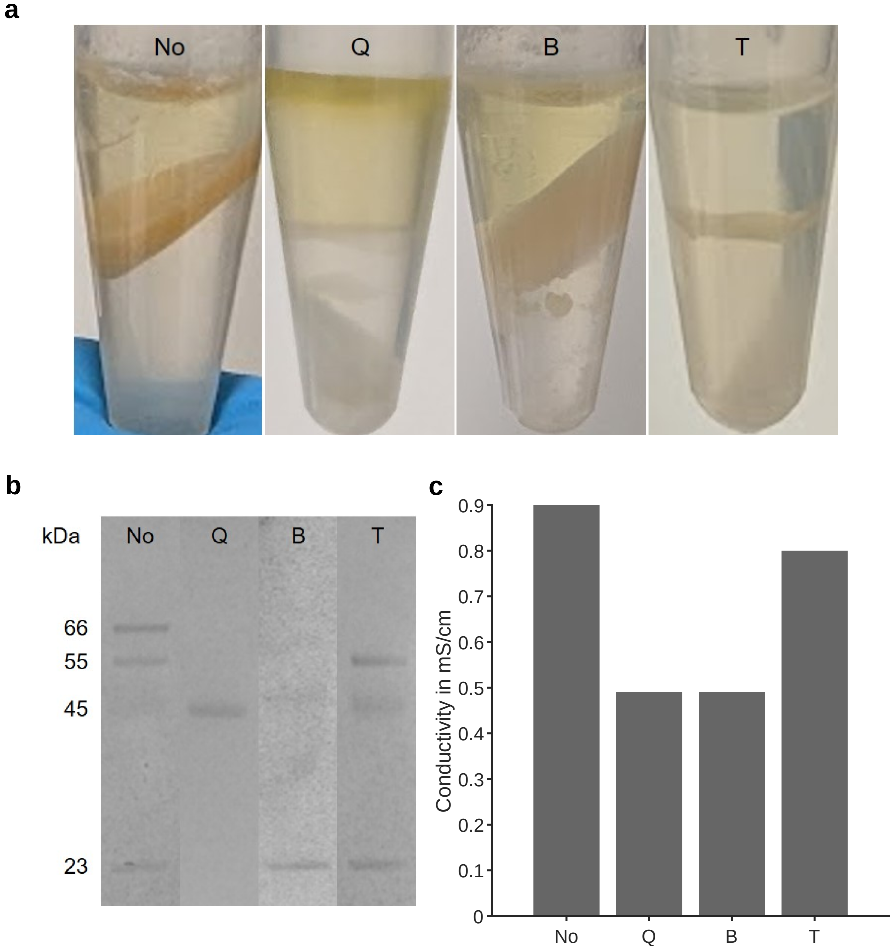

Figure S2. Impact of pre- and post-ATPS capture lysis on ATPS formation, protein partitioning, and sample conductivity. (a) ATPS formation after pre-capture lysis using enzymatic (Q), buffer-based (B), or reducing (T) methods, compared with a non-lysed reference (No). (b) SDS-PAGE analysis of protein present in the bottom phase after post-capture lysis. ATPS composition was 19.5/13.8% (w/w) PEG/salt. Samples were purified using the optimized reverse elution protocol. (c) Conductivity of bottom-phase samples after post-capture lysis under the four conditions indicated. Data is reported for a representative experiment.

TLL	PEG	Salt	Plasma	PEG 1000	NaH_2PO_4	K_2HPO_4	Plasma	BP volume
41% (w/w)	27.9% (w/w)	13.8% (w/w)	66.7% (w/w)	292.5 mg	46.9 mg	159.3 mg	1000 µL	500 µL
34% (w/w)	19.5% (w/w)	8.1% (w/w)	64.0% (w/w)	448.7 mg	29.5 mg	100.0 mg	1000 µL	250 µL
31% (w/w)	30.0% (w/w)	7.0% (w/w)	63.0% (w/w)	490.5 mg	26.1 mg	88.4 mg	1000 µL	185 µL

Table S1. ATPS compositions used for DNA capture in the bottom phase (BP). Tie-line length (TLL), PEG 1000, salt, and plasma contents are given in % (w/w). For a plasma input of 1000 µl, the corresponding masses of PEG and phosphate salts (mg) and the resulting bottom phase volume are reported.

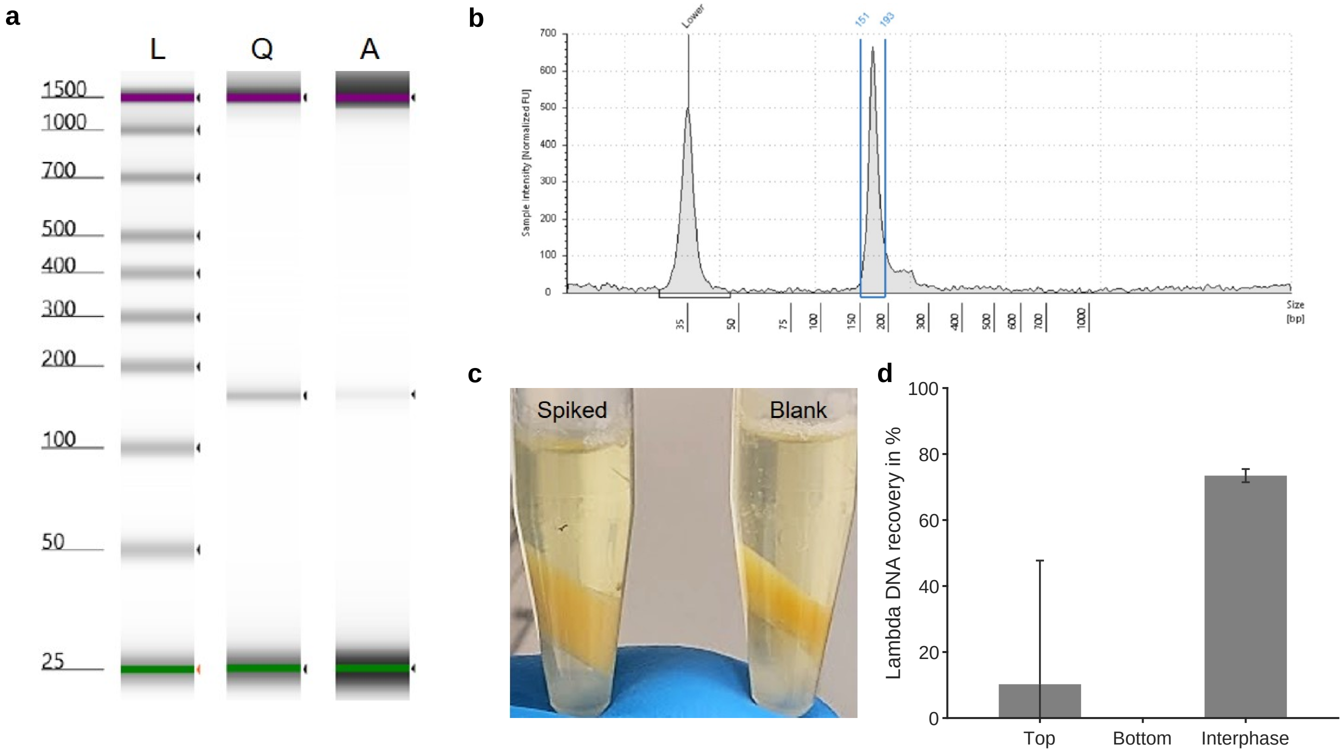

Figure S3. Effect of DNA fragment length on recovery and phase formation. (a) TapeStation analysis of 160 bp DNA extracted from human plasma using the ATPS-based process (A) and the manual silica-based QIAamp Circulating Nucleic Acid Kit (Q). Fragment-length analysis was performed on a representative sample with a D1000 ScreenTape; lane L is the internal ladder. For the ATPS workflow, DNA was recovered from the bottom phase. (b) Electropherogram of 160 bp DNA extracted from human plasma using the ATPS-based process. Analysis was performed on a representative sample with a cell-free DNA ScreenTape. (c) Phase formation of a 27.9/8.1% (w/w) PEG/salt ATPS with and without spiked lambda-DNA. (d) Recovery of lambda-DNA in the top phase, bottom phase, and interphase of the same ATPS. Data represent mean ± SD of technical triplicates.

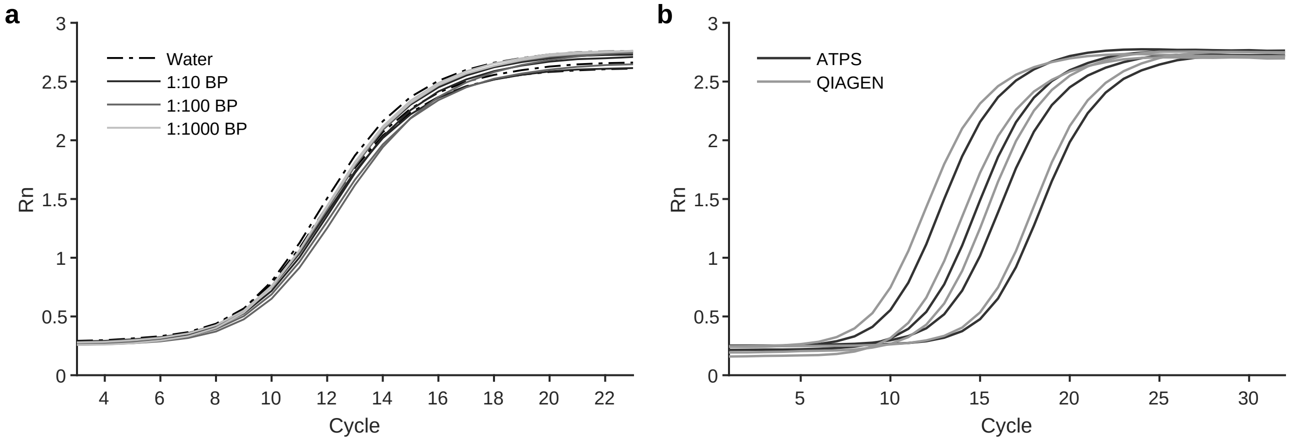

Figure S4. Assessment of qPCR amplification performance and potential matrix inhibition. (a) Amplification curves for 160 bp DNA spiked at 5 ng/mL into water and into a dilution series of the ATPS bottom phase extracts (BP) to evaluate potential matrix inhibition of PCR amplification. Fluorescence intensity is plotted against cycle number. Each sample was measured in technical triplicate, and all amplification curves are shown. (b) Amplification curves for 160 bp DNA spiked at different concentrations and extracted using the ATPS-based workflow or the silica-based QIAGEN kit. Due to differences in recovery efficiency between the extraction methods, DNA concentrations are not identical, and the plot is intended for qualitative comparison of amplification performance rather than direct quantitative comparison. Measurements were performed in technical triplicate, but only one representative curve per sample is shown for clarity.

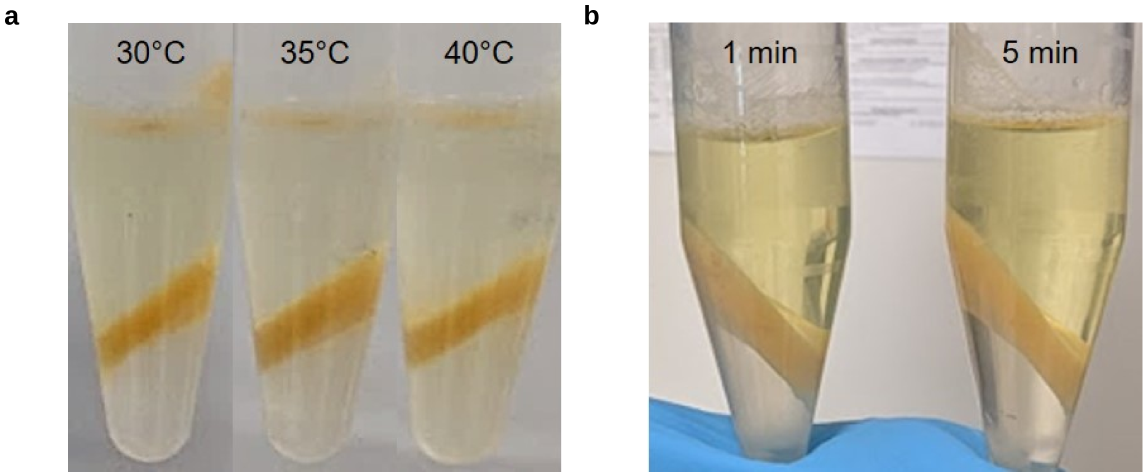

Figure S5. Effect of process parameters on ATPS formation. (a) Phase formation after incubation at different temperatures (30, 35, and 40 °C), followed by centrifugation at 8000 × g for 5 min. (b) Phase formation after centrifugation at 8000 × g for 1 or 5 min, following incubation at room temperature.

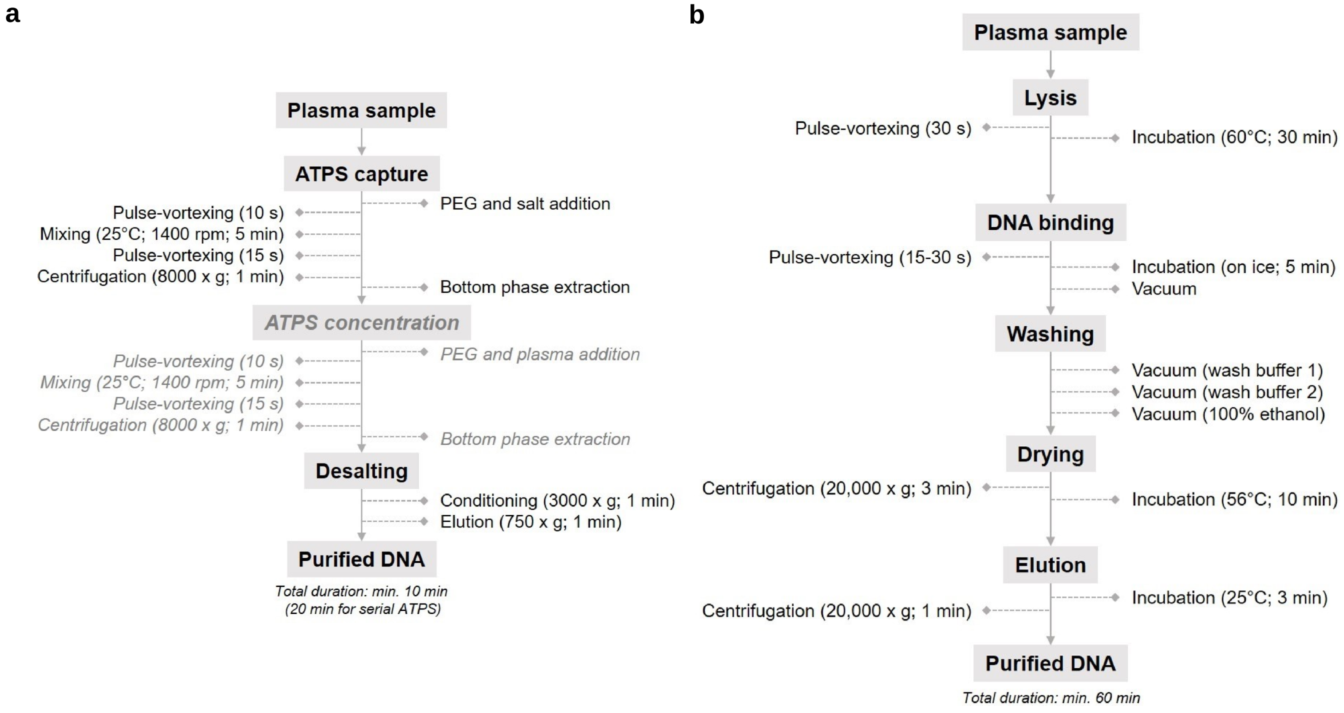

Figure S6. Schematic comparison of (a) the ATPS-based extraction workflow and (b) the QIAamp Circulating Nucleic Acid Kit protocol from plasma sample to purified, sequencing-ready DNA.

Sample	Unique fragments	Unique start sites	On-target dedupl. ratio	Median frag. length	Mean bases coverage	Median bases coverage	Mean targets coverage	Median targets coverage
ATPS 1, spiked	2,783,484	261.81	3.05:1	103 bp	4414.78	3946.00	4027.88	3712.00
ATPS 1, blank	3,466,640	281.51	3.35:1	106 bp	5640.04	5076.00	5079.58	4687.00
ATPS 2, spiked	3,983,902	282.43	2.91:1	108 bp	6588.30	5895.00	5905.71	5524.33
ATPS 2, blank	3,057,152	265.57	2.91:1	104 bp	4945.40	4341.00	4464.17	4080.45

Table S2. Quality metrics of NGS library preparation for ATPS-extracted cfDNA samples. Samples include DNA-free plasma spiked at 5% variant allele frequency (VAF) (spiked) and 0% VAF controls (blank). Reported library quality attributes include the number of unique fragments, the average number of unique start sites per GSP2, the on-target deduplication ratio, the median DNA fragment length, and the mean and median bases and targets coverage for extracts obtained using a single ATPS step (ATPS 1) or two consecutive ATPS steps (ATPS 2). Data is reported for a representative experiment.
